# Sphingosine-1-phosphate receptor 3 promotes leukocyte rolling by mobilizing endothelial P-selectin

**DOI:** 10.1038/ncomms7416

**Published:** 2015-04-02

**Authors:** Claudia Nussbaum, Sarah Bannenberg, Petra Keul, Markus H. Gräler, Cassiano F. Gonçalves-de-Albuquerque, Hanna Korhonen, Karin von Wnuck Lipinski, Gerd Heusch, Hugo C. de Castro Faria Neto, Ina Rohwedder, Joachim R. Göthert, Vysakh Pushpa Prasad, Günter Haufe, Baerbel Lange-Sperandio, Stefan Offermanns, Markus Sperandio, Bodo Levkau

**Affiliations:** 1 Walter Brendel Center, Ludwig Maximilians Universität München, 81377 München, Germany; 2 Dr v. Haunersches Children’s Hospital, Ludwig Maximilians University München, 80337 München, Germany; 3 Institute of Pathophysiology, West German Heart and Vascular Center, University Hospital Essen, University of Duisburg-Essen, 45122 Essen, Germany; 4 Department of Anesthesiology and Intensive Care Medicine, Center for Sepsis Control and Care, Center for Molecular Biomedicine, University Hospital Jena, 07745 Jena, Germany; 5 Laboratorio de Imunofarmacologia, Instituto Oswaldo Cruz, Fiocruz, Rio de Janeiro 21040900, Brazil; 6 Max Planck Institute for Heart and Lung Research, 61231 Bad Nauheim, Germany; 7 Department of Hematology, University Hospital Essen, University of Duisburg-Essen, 45122 Essen, Germany; 8 Organisch-Chemisches Institut, Westfälische Wilhelms-Universität Münster, 48149 Münster, Germany

## Abstract

Sphingosine-1-phosphate (S1P) participates in inflammation; however, its role in leukocyte rolling is still unclear. Here we use intravital microscopy in inflamed mouse cremaster muscle venules and human endothelial cells to show that S1P contributes to P-selectin-dependent leukocyte rolling through endothelial S1P receptor 3 (S1P_3_) and Gα_q_, PLCβ and Ca^2+^. Intra-arterial S1P administration increases leukocyte rolling, while S1P_3_ deficiency or inhibition dramatically reduces it. Mast cells involved in triggering rolling also release S1P that mobilizes P-selectin through S1P_3_. Histamine and epinephrine require S1P_3_ for full-scale effect accomplishing it by stimulating sphingosine kinase 1 (Sphk1). In a counter-regulatory manner, S1P_1_ inhibits cAMP-stimulated Sphk1 and blocks rolling as observed in endothelial-specific *S1P*_*1*_^−/−^ mice. In agreement with a dominant pro-rolling effect of S1P_3_, FTY720 inhibits rolling in control and *S1P*_*1*_^−/−^ but not in *S1P*_*3*_^*−/−*^ mice. Our findings identify S1P as a direct and indirect contributor to leukocyte rolling and characterize the receptors mediating its action.

## Abstract (web summary)

The lipid sphingosine-1-phosphate (S1P) is known to mediate leukocyte recruitment in inflammation. Here, Nussbaum *et al.* show that S1P, via its receptor S1P3, also regulates leukocyte rolling on endothelium by promoting the presentation of the adhesion molecule P-selectin on the endothelial surface.

## Graphical abstract

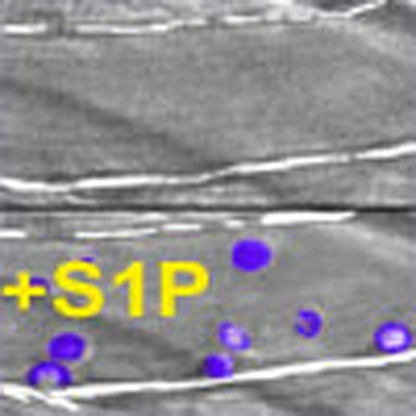

Sphingosine-1-phospate (S1P) is a bioactive lysophospholipid with important functions in the immune and cardiovascular systems and has been implicated in different aspects of inflammatory disorders^1^. High S1P concentrations have been measured locally at inflammatory sites^2^,^3^, where it is generated by sphingosine kinases activated through inflammatory cytokines, lipopolysaccharide (LPS) or thrombin. This suggests a role for S1P in the propagation of the inflammatory response^4^. In contrast, S1P opposes the pathologically increased endothelial permeability common to all inflammatory processes and prevents vascular leakage caused by LPS, thrombin or platelet-activating factor^5^,^6^,^7^. There is also evidence both for stimulatory and inhibitory effects of S1P on leukocyte recruitment in inflammation. In favour of a pro-inflammatory role, exogenous S1P induces endothelial vascular cell adhesion molecule 1 (VCAM-1) and E-selectin, while endogenous S1P mediates the stimulatory effect of tumor-necrosis factor (TNF)-α and LPS on adhesion molecules, which is suppressed by S1P_1_ short interfering RNA^8^,^9^,^10^. In support, chronic overexpression of sphingosine kinase 1 augments VCAM-1 and E-selectin expression and enhances neutrophil adhesion after TNF-α^11^. In contrast, both S1P and S1P_1_ agonists have been demonstrated to inhibit TNF-α-induced endothelial adhesion molecule expression and adherence of inflammatory cells by interfering with endothelial NF-κB and stimulating nitric oxide production^12^,^13^,^14^. Several explanations have been put forward for these apparent discrepancies such as the assumption that S1P receptors may be differentially expressed among endothelial beds, and that the wide range of S1P concentrations employed in the individual studies may have led to contrary effects as observed for S1P in other systems^15^. However, in all these studies the focus has been on S1P_1_, while any involvement of S1P receptor 3 (S1P_3_) has not been addressed.

In contrast to the effects of S1P on firm leukocyte adhesion, its role on leukocyte rolling—the crucial initial step of inflammatory cell recruitment—has not been investigated in detail. Recently, sphingosine kinase-1 has been shown to contribute to histamine-induced leukocyte rolling, but the mechanism of action has remained elusive^16^. In general, leukocyte capture and rolling is mediated by the selectin family of adhesion molecules consisting of three selectins: L-, E- and P-selectin^17^,^18^. L-selectin is expressed on most leukocytes and mediates leukocyte capture and rolling through binding to selectin ligands found on high endothelial venules of lymphoid organs. E-selectin and P-selectin are expressed on the activated vascular endothelium during inflammation and mediate rolling through binding to carbohydrate moieties of selectin ligands on leukocytes^17^,^18^. Among the selectins, P-selectin contributes to disease pathology in many experimental models including myocardial and renal infarction, thrombosis, stroke, atherosclerosis, cerebral malaria and sickle cell disease^19^, as has been shown using P-selectin-blocking antibodies and knockout strategies in mouse, feline and baboon models^20^,^21^,^22^,^23^.

We have recently identified leukocyte recruitment defects in *S1P*_*3*_^*−/−*^ mice in atherosclerosis and peritoneal inflammation that were caused by both haematopoietic and non-haematopoietic S1P_3_ deficiency^3^. In addition, treatment with the S1P analogue FTY720 severely reduced leukocyte recruitment into the inflamed peritoneum^3^. Currently, it is controversial if S1P in general and S1P_3_ in particular affect leukocyte rolling.

In this study, we address this issue by studying P-selectin-dependent leukocyte rolling in surgically prepared post-capillary venules of the mouse cremaster muscle *in vivo*. These studies are complemented by experiments in a novel *in vitro* system to investigate the mechanisms of the rapid P-selectin mobilization that occurs within minutes in endothelial cells and stems from the exocytosis of Weibel–Palade bodies. Our results demonstrate a unique role of S1P and S1P_3_ in P-selectin-dependent rolling both by direct action as well as by contributing to P-selectin mobilization by other agonists.

## Results

### S1P_3_ deficiency and inhibition reduce leukocyte rolling

**Figure 1:**
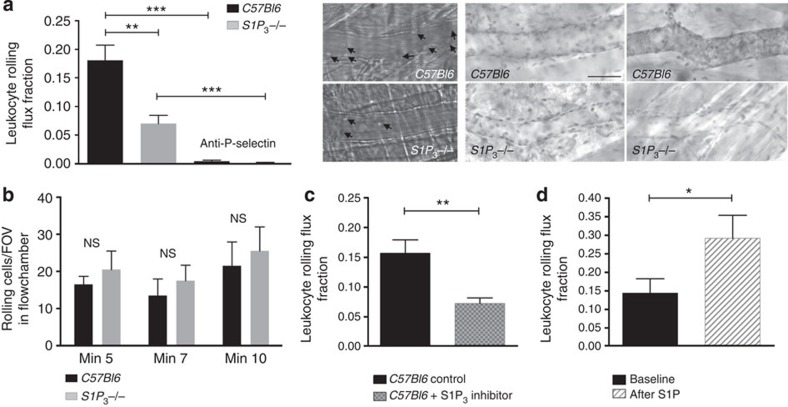
Leukocyte rolling is reduced in *S1P*_*3*_^*−/−*^ mice and after S1P_3_ blockade, respectively, and is enhanced by S1P administration. Leukocyte rolling was quantified in postcapillary venules of the cremaster muscle of (**a**) *S1P*_*3*_^*−/−*^ mice (*n*=5) and *C57Bl6* controls (*n*=6). Rolling was abolished by systemic injection of a P-selectin-blocking antibody in both groups. Middle: representative snap shot images of rolling leukocytes (arrows) are shown. Right: representative whole-mount immunohistochemistry for P-Selectin after *in vivo* injection of an anti-P-selectin antibody (left: no staining in uninjured cremaster, right: luminal P-selectin staining in the contralateral exteriorized cremaster). Scale bar, 40 μm. (**b**) Unaltered rolling of *S1P*_*3*_^*−/−*^ leukocytes on immobilized P-selectin. Microflow chambers were coated with recombinant murine P-selectin and perfused with arterial blood diverted from the carotid artery of *C57Bl6* and *S1P*_*3*_^*−/−*^ mice (*n*=3 each) for the indicated times. The number of rolling cells was quantified per field of view (FOV). (**c**) Rolling in *C57Bl6* mice with (*n*=4) and without (*n*=5) the S1P_3_ inhibitor TY-52156 (1.25 mg kg^−1^ intraperitoneally (i.p.) 30 min before experiment). (**d**) Rolling in *C57Bl6* mice at baseline and immediately after S1P injection into the carotid artery (30 μg kg^−1^, *n*=5). Quantitative data are presented as mean±s.e.m. Significance was established using a Wilcoxon rank sum test or a paired two-tailed Student’s *t*-test. In all experiments: *P<0.05; **P<0.01; ***P<0.001.

To examine whether S1P_3_ plays a role in P-selectin-dependentleukocyte rolling *in vivo*, we employed intravital microscopy to monitor rolling in post-capillary venules of the surgically exteriorized mouse cremaster muscle^24^,^25^. In this model, profound spontaneous leukocyte rolling is induced by the surgical preparation of the cremaster (hence termed trauma), which exclusively depends on the rapid mobilization of P-selectin from endothelial Weibel–Palade bodies to the luminal surface in its initial phase (<45 min after exteriorization of the cremaster muscle)^17^,^26^,^27^,^28^. Elegant studies from the 1990s have shown that the endothelial P-selectin mobilization is in large part caused by activation products of tissue mast cells released upon mechanical manipulation during surgery^29^. Here we employed this model to study leukocyte rolling in *S1P*_*3*_^*−/−*^ mice and observed that their rolling flux fraction (as a measure of rolling) was greatly diminished compared with C57Bl6 controls (Fig. 1a). Neither microvascular/haemodynamic conditions nor leukocyte rolling velocities differed between groups excluding any bias by these variables (Table 1 and Supplementary Fig. 1). Systemic application of a P-selectin-blocking antibody completely abrogated leukocyte rolling in both genotypes (Fig. 1a) confirming the dependence of rolling on P-selectin in this model and both genotypes, respectively. In addition, whole-mount immunohistochemistry for intravascular P-selectin clearly showed the appearance of luminal P-selectin in controls but not in *S1P*_*3*_^*−/−*^ mice (Fig. 1a). Of note, S1P_3_-deficient leukocytes had no intrinsic defect in rolling as we determined in an *ex vivo* flow chamber system^30^, where blood was diverted from the carotid artery into microflow chambers coated with immobilized murine P-selectin^31^ (Fig. 1b). We also assessed cell surface expression of P-selectin glycoprotein ligand-1 as the main, if not only, functional P-selectin ligand on neutrophils, and found no differences between *S1P*_*3*_^*−/−*^ and *C57Bl6* mice (Supplementary Table 1).

We next tested the effect of TY-52156, a highly specific S1P_3_ inhibitor^32^, on leukocyte rolling in C57Bl6 mice. As TY-52156 is not commercially available, we synthesized it according to published protocols^32^ and confirmed its specificity for S1P_3_ in comparison to S1P_1_ (Supplementary Figs 2,3). Intraperitoneal administration of TY-52156 (1.25 mg per kg body weight) 30 min before the experiment leads to a dramatic reduction in leukocyte rolling (Fig. 1c). To test whether the opposite–stimulation of the vasculature by exogenous S1P–would increase rolling, we injected S1P directly into the carotid artery (30 μg per kg body weight) in *C57Bl6* mice and measured leukocyte rolling immediately thereafter. Indeed, we observed that rolling was dramatically enhanced by S1P (Fig. 1d).

### S1P_3_ induces P-selectin and rolling via G_q_, PLC and Ca^2+^

**Figure 2:**
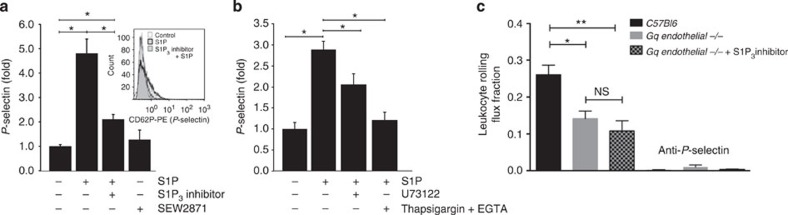
S1P mobilizes P-selectin in endothelial cells and induces rolling through a S1P_3_/G_q_/PLC/Ca^2+^ pathway. (**a**) HUVECs were stimulated with S1P (1 μM) for 5 min in the presence or absence of the S1P_3_ inhibitor TY-52156 (10 μM) or stimulated with the S1P_1_ agonist SEW2871 (1 μM). P-selectin surface expression was analysed using flow cytometry. Data shown are from at least three independent experiments. Representative histogram is shown as inset. (**b**) HUVECs were stimulated with S1P (1 μM) in the presence or absence of U73122 (3 μM for 2 min) or thapsigargin (3 μM for 5 min) in combination with EGTA (3 mM for the last 1 min). P-selectin was analysed using flow cytometry 5 min after the addition of S1P. Data shown are from at least three independent experiments. (**c**) Leukocyte rolling was quantified in postcapillary venules of the cremaster muscle of *G*_*q/11*_*−/−* mice (Gq endothelial −/−) in the presence (*n*=4) or absence (*n*=5) of the S1P_3_ inhibitor TY-52156 (1.25 mg kg^−1^ i.p. 30 min before the experiment) and in controls (*n*=4). P-selectin-blocking antibody was injected to confirm complete dependence of rolling on P-selectin. Quantitative data are presented as mean±s.e.m. Significance was established using a paired two-tailed Student’s *t*-test or a Kruskal–Wallis analysis of variance (ANOVA) on ranks. *P<0.05; **P<0.01.

Exposure of cell surface P-selectin on luminal endothelial cells is the prerequisite for trauma-induced leukocyte rolling in the experimental model we have used here^17^,^26^,^27^,^28^. To pursue the mechanism by which S1P and S1P_3_ affect P-selectin mobilization, we established an assay for quantitative assessment of cell surface P-selectin using flow cytometry in human umbilical endothelial cells (HUVECs) as the only system applicable for such studies. Nevertheless, HUVECs are still extremely difficult to work with in respect to studying P-selectin as they express it only under certain culture conditions and lose ability for mobilizing it already after one to two passages. Nevertheless, we successfully established the assay and validated it extensively using histamine and H1 and H2 receptor antagonists (Supplementary Fig. 4). Using this assay, we observed that stimulation of HUVEC with 1 μM S1P resulted in an extremely rapid (within 5 min) appearance of P-selectin on the cell surface (Fig. 2a). In contrast, the agonist of the S1P_1_ receptor SEW2871 was ineffective (Fig. 2a). In agreement with a role of S1P_3_ in the process, preincubation with 10 μM TY-52156 for 30 min substantially diminished P-selectin mobilization by S1P (Fig. 2a).

We then addressed the signalling pathways downstream of S1P/S1P_3_ responsible for P-selectin mobilization. Phospholipase C (PLC) and Ca^2+^ are known to play a role in the exocytosis of Weibel–Palade bodies^33^, and S1P has been shown to activate PLC in CHO cells overexpressing S1P_3_ (ref. 34, 35). Thus, we added the PLC inhibitor U73122 (3 μM) 2 min before S1P stimulation and observed a clear reduction in P-selectin mobilization (Fig. 2b). To address the role of Ca^2+^, we depleted internal Ca^2+^ pools by adding thapsigargin (3 μM) for 5 min before stimulation with S1P, while simultaneously preventing the extracellular Ca^2+^ influx by EGTA (3 mM) added for the last minute. This treatment substantially suppressed P-selectin mobilization (Fig. 2b).

Although S1P_3_ couples to three G proteins (G_q_, G_i_ and G_12/13_), the predominant signalling pathway it exercises is through G_q_ (ref. 36). To test for an involvement of G_q_ in leukocyte rolling in our model in general, we employed mice deficient for G_q_ in endothelial cells on a global G_11_-deficient background (*G*_*q/11*_*−/−* mice)^37^. We observed them to have a substantial reduction in leukocyte rolling (Fig. 2c). Administration of TY-52156 did not reduce rolling any further in *G*_*q/11*_*−/−* mice (Fig. 2c), suggesting that under normal circumstances S1P_3_ enhances rolling through G_q_.

### FTY720 and S1P lyase blockade suppress rolling by impacting on S1P_3_

**Figure 3:**
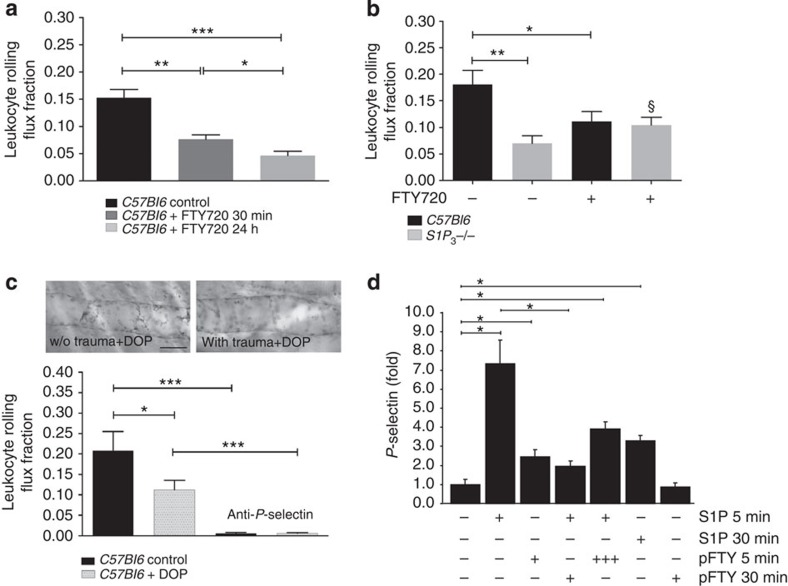
FTY720 inhibit leukocyte rolling and P-selectin mobilization by S1P by interfering with S1P_3_. Reduced rolling after S1P lyase inhibition. (**a**) Leukocyte rolling in *C57Bl6* mice treated without (*n*=5) or with 1.25 mg kg^−1^ FTY720 i.p. either 30 min or 24 h (*n*=6 each) before experiment. (**b**) Leukocyte rolling in *C57Bl6* (*n*=8) and *S1P*_*3*_^*−/−*^ mice (*n*=5) treated with 1.25 mg kg^−1^ i.p. FTY720 30 min before experiment. Untreated *C57Bl6* and *S1P*_*3*_^*−/−*^ mice served for comparison (*n*=6 and 5). (**c**) Leukocyte rolling in *C57Bl6* mice treated with (*n*=5) and without the S1P lyase inhibitor DOP (*n*=4). Top: whole-mount immunohistochemistry for P-Selectin after *in vivo* injection of an anti-P-selectin antibody shows the lack of P-selectin mobilization in the exteriorized traumatized cremaster (right) compared with the contralateral uninjured one (left). Scale bar, 40 μm. (**d**) HUVECs were stimulated with S1P (1 μM) or pFTY720 (1 μM) for 5 and 30 min with and without pFTY720 preincubation (1 μM) for 30 min. In some experiments, pFTY720 (10 μM) and S1P (1 μM) were added simultaneously for 5 min. ‘+++’ indicates the 10-fold higher pFTY720 concentration that was added simultaneously with S1P in this case. P-selectin surface expression was analysed by flow cytometry. Data are from three independent experiments. Quantitative data are presented as mean±s.e.m. Significance was established using a Kruskal–Wallis ANOVA on ranks or a Wilcoxon rank sum test, or a paired two-tailed Student’s *t*-test (**d**). *P*<0.05; **P<0.01; ***P<0.001; §<0.05 between *S1P*_*3*_^*−/−*^ and *S1P*_*3*_^*−/−*^ with FTY720 in (**b**).

On the basis of our results so far, we hypothesized that downregulation of S1P_3_ by FTY720 would inhibit leukocyte rolling to a similar extent as does S1P_3_ deficiency or a S1P_3_ inhibitor. Furthermore, if S1P_3_ were the only receptor inducing P-selectin mobilization by S1P, FTY720 should not decrease rolling any further in *S1P*_*3*_^*−/−*^ mice. Indeed, administration of 1.25 mg per kg FTY720 either 30 min or 24 h before the experiment dramatically attenuated leukocyte rolling (Fig. 3a). In agreement with our second hypothesis, we observed no additional inhibition of rolling by FTY720 in *S1P*_*3*_^*−/−*^ mice (Fig. 3b). Finally, treatment with the S1P lyase inhibitor 4-deoxypyridoxine (DOP) that elevates plasma and tissue S1P and downregulates all S1P receptors^38^ also inhibited rolling (Fig. 3c). Rolling velocities did not differ significantly between the experimental groups (Supplementary Fig. 1), and P-selectin-mediated rolling was independent of putative DOP effects on plasma or leukocytes as no differences in rolling were observed on immobilized P-selectin (Supplementary Fig. 5).

We then went further to examine the effect of FTY720 on P-selectin mobilization *in vitro*. Pretreatment of HUVEC with the active, phosphorylated form of FTY720 (pFTY720, 10 μM for 30 min) almost completely abolished S1P-induced mobilization of P-selectin (Fig. 3d). PhosphoFTY720 itself (1 μM) had only a minor, almost negligible effect on P-selectin compared with 1 μM S1P after 5 min and no effect at all after 30 min (a time when the S1P effect was still clearly visible; Fig. 3d). This may seem surprising in light of the agonistic effect of pFTY720 known for all S1P receptors except S1P_2_ before their subsequent cell surface downregulation. However, we have previously shown that pFTY720 activates only the G_i_ and not the G_q_ signalling pathway of S1P_3_ and also inhibits G_q_-transmitted S1P_3_ signalling by native S1P^39^. Here we observed something very similar in respect to P-selectin: addition of a 10-fold higher concentration of pFTY720 simultaneously with S1P resulted in a clear inhibition of S1P-induced P-selectin mobilization (Fig. 3d). In agreement, addition of S1P or pFTY720 for 30 min to another cellular system — S1P_3_-overexpressing CHO cells — followed by removal and subsequent (30 min later) S1P stimulation abolished S1P_3_-dependent signalling such as Erk MAPK activation (Supplementary Fig. 6).

### S1P_3_ conveys part of the P-selectin mobilization by histamine

**Figure 4:**
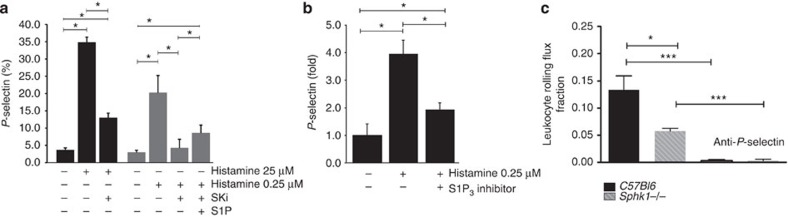
S1P_3_ and Sphk1 mediate in part the effect of histamine on P-selectin mobilization and leukocyte rolling. (**a**) HUVECs were stimulated with two histamine concentrations (0.25 and 25 μM) in the presence or absence of SKi (25 μM) or SKi+S1P (1 μM) for 5 min as indicated, and cell surface P-selectin was measured by flow cytometry. Data shown are from three independent experiments. (**b**) HUVECs were stimulated with histamine (0.25 μM) in the presence or absence of the S1P_3_ inhibitor TY-52156 (10 μM) and P-selectin was analysed by flow cytometry. Data are from five independent experiments. (**c**) Leukocyte rolling was quantified in postcapillary venules of the cremaster muscle of *Sphk1*^*−/−*^ mice and controls (*n*=4 per group). P-selectin-blocking antibody was injected to confirm rolling dependence on P-selectin. Quantitative data are presented as mean±s.e.m. Significance was established using a paired two-tailed Student’s *t*-test or a Wilcoxon rank sum test.*P<0.05; *P<0.05; ***P<0.001.

P-selectin mobilization and consecutive leukocyte rolling in the trauma-stimulated cremaster muscle venules that we have examined here are known to be predominantly mediated by products of tissue mast cells activated upon mechanical manipulation during surgery^29^. Of these products, histamine accounts for approximately half of the effect on rolling as elegantly shown using H1 and H2 histamine receptor antagonists^29^. Recently, Sun *et al.*^16^ demonstrated that histamine superfusion of the cremaster muscle induces leukocyte rolling dependent on sphingosine kinase 1 (Sphk1), but the mechanism has remained unidentified. We asked the question whether S1P_3_ might be involved in histamine-induced P-selectin mobilization. To tackle this, we first titrated down the histamine concentration from 25 μM (routinely used in the vast majority of the literature) to a 100-fold lower concentration of 0.25 μM (Fig. 4a). This much lower histamine concentration still leads to a substantial P-selectin mobilization in HUVEC within 5 min (Fig. 4a). The well-characterized sphingosine kinase inhibitor 4-[[4-(4-chlorophenyl)-2-thiazolyl]amino]-phenol (SKi, 25 μM (ref. 40)) suppressed P-selectin mobilization at both histamine concentrations, but was particularly effective at the lower one (Fig. 4a). Remarkably, administration of exogenous S1P in addition to SKi partially restored P-selectin mobilization by histamine (Fig. 4a), arguing for a S1P receptor conveying Sphk1-mediated, histamine-dependent P-selectin mobilization. We then tested whether the responsible S1P receptor may be S1P_3_. Indeed, preincubation with the S1P_3_ inhibitor TY-52156 for 30 min dramatically reduced P-selectin mobilization by 0.25 μM histamine (Fig. 4b). Thus, the reduction of histamine to lower and presumably more physiological concentrations in our model allowed us to assign part of its P-selectin-mobilizing effect to the engagement of S1P_3_ through endogenous Sphk1-generated S1P. Finally, we observed that leukocyte rolling was substantially reduced in cremaster muscle venules of *Sphk1*^*−/−*^ mice *in vivo* compared with *C57Bl6* controls (Fig. 4c). Altogether, our data suggest that part of the histamine effect on P-selectin-mediated rolling is mediated by its activation of Sphk1, production of endogenous S1P and subsequent stimulation of P-selectin mobilization by endothelial S1P_3_.

### cAMP induces P-selectin and rolling through Sphk1 and S1P_3_

**Figure 5:**
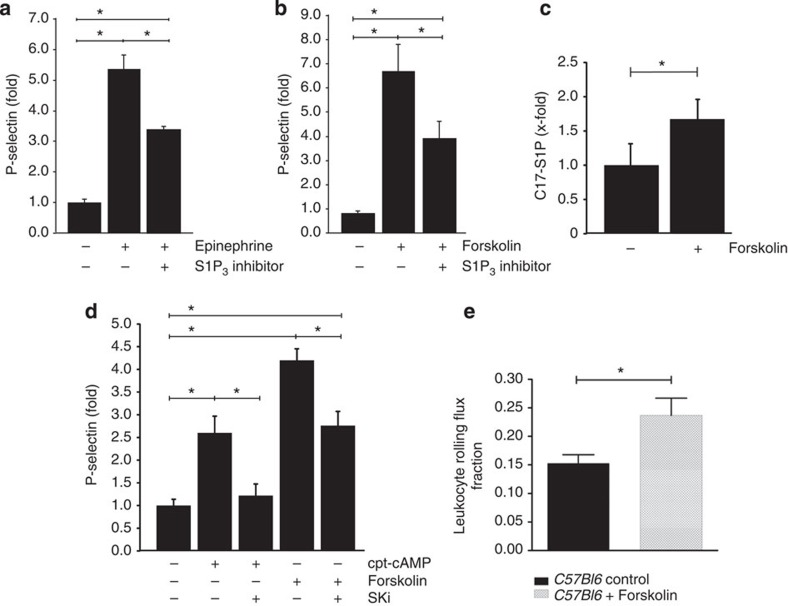
Epinephrine and forskolin stimulate P-selectin mobilization and leukocyte rolling through adenylate cyclase/cAMP-dependent Sphk1 activation and engagement of S1P_3_. HUVECs were stimulated with (**a**) epinephrine (9 μM) in the presence of IBMX (100 μM) and (**b**) forskolin (10 μM) with or without the S1P_3_ inhibitor TY-52156 (10 μM), and P-selectin was analysed by flow cytometry. Data shown are from five independent experiments. (**c**) HUVECs were stimulated with forskolin (10 μM) in the presence of C17-sphingosine for 30 min, washed and analysed for intracellular C17-S1P using mass spectrometry. Data shown are from four independent experiments. (**d**) HUVECs were stimulated with forskolin (10 μM) or cpt-cAMP (0.3 mM) in the presence or absence of SKi (25 μM) for 30 min. P-selectin was analysed by flow cytometry. Data shown are from five independent experiments. (**e**) Leukocyte rolling in *C57Bl6* mice treated with forskolin or vehicle (*n*=5 per group). Quantitative data are presented as mean±s.e.m. Significance was established using a paired two-tailed Student’s *t*-test or a Wilcoxon rank sum test. *P<0.05.

Several other agents besides histamine such as thrombin, vasopressin receptor agonists, purine nucleotides and epinephrine are well known to induce rapid P-selectin mobilization^41^. Many of them elevate intracellular cAMP through activation of the G_s_/adenylate cyclase (AC) pathway; however, the exact mechanisms leading to subsequent P-selectin mobilization are not entirely clear. In our hands, epinephrine (9 μM) potently induced P-selectin mobilization in HUVEC (Fig. 5a). Remarkably, the S1P_3_ inhibitor TY-52156 substantially albeit not entirely suppressed epinephrine-induced P-selectin mobilization (Fig. 5a). Assuming that the cAMP/AC pathway is involved, we applied forskolin (10 μM) as an established tool to directly stimulate AC. Indeed, forskolin potently mobilized P-selectin mobilization in HUVEC (Fig. 5b). Interestingly, this was clearly inhibited by S1P_3_ inhibitor TY-52156 (Fig. 5b). These data suggested that a mechanism involving AC activation and relying on S1P_3_ is in part responsible for P-selectin mobilization by cAMP-generating agents such as epinephrine and forskolin. Forskolin has been shown to activate Sphk1 in pheochromocytoma cells^42^. We thus hypothesized that this may be the case in endothelial cells as well. Thus, we determined enzymatic Sphk1 activity after forskolin stimulation by measuring the conversion of C17-sphingosine to C17-S1P using mass spectrometry. Indeed, C17-S1P production was clearly increased after forskolin (10 μM) treatment of HUVEC for 30 min (Fig. 5c). To explore the consequences for P-selectin mobilization, we employed forskolin and the nondegradable cAMP analogue cpt-cAMP, respectively, to activate Sphk and added the Sphk inhibitor SKi. Indeed, both forskolin (10 μM) and cpt-cAMP (0.3 mM) induced P-selectin mobilization, and in both cases SKi treatment substantially reduced it (Fig. 5d). Finally, we sought to reproduce these findings at the level of leukocyte rolling *in vivo* and applied forskolin (1.3 mg kg^−1^) intraperitoneally in our cremaster model. In agreement with its induction of P-selectin *in vitro*, forskolin clearly stimulated leukocyte rolling *in vivo* (Fig. 5e). In summary, these data have identified a novel cAMP-dependent mechanism of P-selectin mobilization that acts through Sphk1 activation and employs subsequent S1P_3_ signalling.

### Mast cell-derived S1P induces P-selectin in HUVEC via S1P_3_

**Figure 6:**
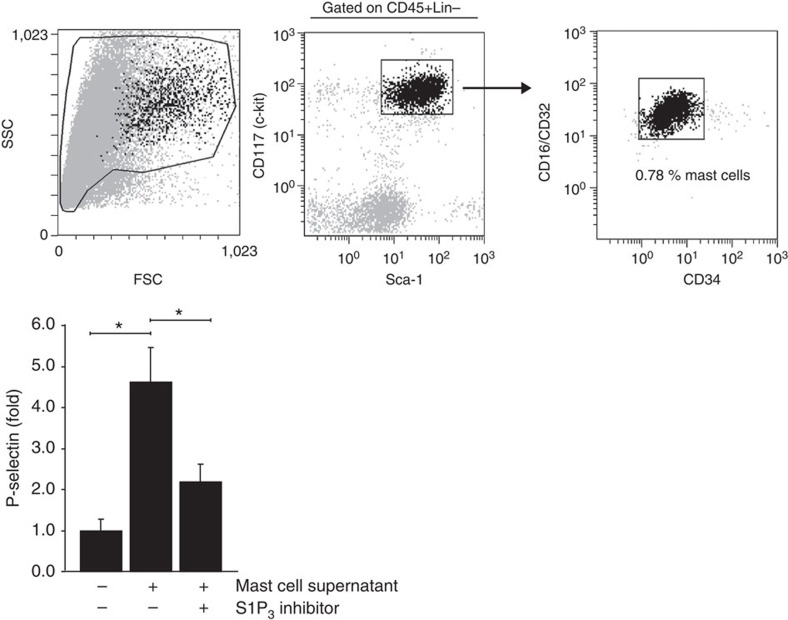
Supernatants of activated mast cells stimulate P-selectin mobilization in HUVEC in a S1P_3_-dependent manner. (Top) FACS analysis of mouse peritoneal mast cells (CD45^+^ Lin^−^ CD117^+^ Sca-1^+^ CD16/32^+^ CD34^+^) used for stimulation. (Bottom) HUVECs were stimulated with supernatants of heat-activated peritoneal mast cells for 10 min in the presence or absence or the S1P_3_ inhibitor TY-52156 (10 μM). Diphenhydramine and cimetidine (10 μM each) were present in all experiments. P-selectin was measured using flow cytometry. Quantitative data are presented as mean±s.e.m. for three independent mast cell preparations and three independent HUVEC preparations. Significance was established using a paired two-tailed Student’s *t*-test. *P*<0.05.

According to previous studies, histamine release from mast cells accounts for half of the leukocyte rolling in the cremaster model that by itself is completely mast cell-dependent^29^. Although S1P is not contained in mast cell granules, mast cell can release large amounts of S1P when allergically stimulated^43^,^44^,^45^. Thus, we hypothesized that tissue mast cells may be another source of S1P in our model thereby causing P-selectin mobilization and rolling. Physical stimuli such as pressure and temperature are known to induce mast cell activation *in vitro* and *in vivo*^46^,^47^. As surrogate for the physical stimulus of mechanical mast cell manipulation occurring during cremaster muscle exteriorization, we employed transient exposure of isolated peritoneal mast cells to a temperature of 53 °C for 2 min as commonly employed to activate mast cell without adverse effects on viability^46^,^47^. We then added the mast cell supernatants immediately to HUVEC and measured P-selectin surface expression (Fig. 6). To exclude effects of histamine released under these conditions, we added diphenhydramine and cimetidine in concentrations sufficient to completely block P-selectin mobilization by 0.25 μM histamine (Supplementary Fig. 4). The mast cell supernatants induced an impressive four- to fivefold increase in cell surface P-selectin expression compared with supernatants of unchallenged mast cells after 10 min (Fig. 6). Remarkably, this increase was suppressed by more than half by the S1P_3_ inhibitor TY-52156 (Fig. 6). Mass spectrometry of the identical supernatants revealed that S1P had been released (90±41 pmol S1P per 10^6^ mast cells) resulting in a 64.73±7.95 nM S1P concentration in the mast cell supernatants that the HUVEC had been exposed to. Another proof of biologically active S1P being present in the mast cell supernatants was their ability to activate Erk MAPK in S1P_1_-overexpressing CHO cells and the inhibition thereof by a S1P_1_ antagonist (Supplementary Fig. 7).

### Rolling is increased in endothelial S1P_1_ deficiency

**Figure 7:**
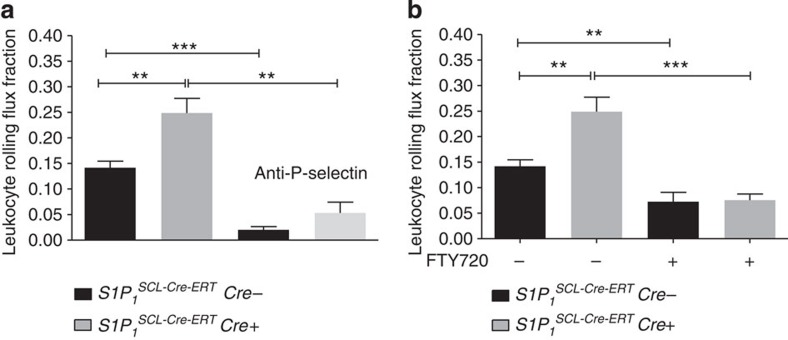
Leukocyte rolling is increased in endothelial-specific S1P_1_ knockout mice (*S1P*_*1*_^*SCL-Cre-ERT*^). (**a**) Leukocyte rolling was quantified in postcapillary venules of the cremaster muscle of *S1P*_*1*_^*SCL-Cre-ERT*^ mice (Cre+ and Cre−, *n*=9 per group). Rolling was also measured after systemic injection of a P-selectin-blocking antibody (*n*=4 per group). (**b**) Leukocyte rolling after administration of 1.25 mg kg^−1^ i.p. FTY720 30 min before the experiment in both genotypes (Cre+ *n*=4, Cre− *n*=3). Quantitative data are presented as mean±s.e.m. Significance was established using a Wilcoxon rank sum test. *P<0.05; *P<0.05; ***P<0.001.

Finally, we examined leukocyte rolling in mice deficient for the endothelial S1P_1_ receptor (*S1P*_*1*_^*SCL-Cre-ERT*^) and observed it to be increased compared with respective controls (Fig. 7a). Furthermore, administration of FTY720 reduced rolling in *S1P*_*1*_^*SCL-Cre-ERT*^ mice to the same lower than basal levels observed with FTY720 in control mice and *C57Bl6* mice, respectively, and *S1P*_*3*_^*−/−*^ mice (Fig. 7b). Microvascular and haemodynamic parameters as well as rolling velocities were unaffected (Table 1 and Supplementary Fig. 1). Interestingly, the highly specific S1P_1_ agonist AUI 954 (ref. 48) inhibited forskolin-induced C17-S1P generation in HUVEC *in vitro* along with inhibiting cAMP generation (Supplementary Fig. 8). These results suggest that S1P_1_ negatively regulates cAMP-stimulated Sphk1 and by reducing S1P generation possibly inhibits P-selectin-dependent rolling, hence acting as an opponent to S1P_3_.

## Discussion

**Figure 8:**
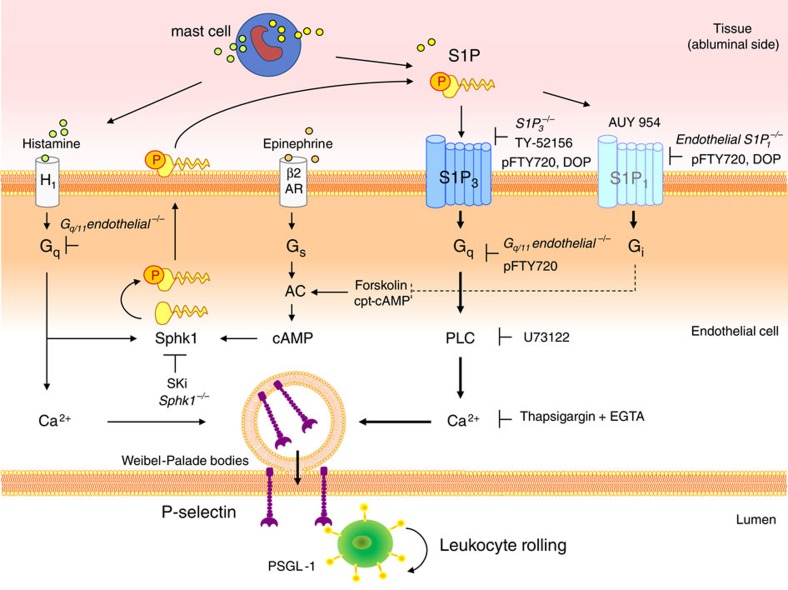
The role of S1P and its receptors in P-Selectin mobilization. Schematic representation of the direct and indirect roles S1P plays in P-selectin mobilization. Mast cells release both histamine and S1P that can both mobilize P-selectin independently through the H1 and S1P_3_ receptor, respectively. However, histamine achieves full scale P-selectin mobilization by additional Gq-dependent activation of Sphk1, S1P production/release and subsequent activation of S1P3. Other P-selectin mobilizing agents such as epinephrine that require cAMP for P-selectin mobilization also employ Sphk1 for full-blown effect but do so through a Gs/AC/cAMP-dependent pathway. S1P1 plays an inhibitory effect on P-selectin-dependent rolling by reducing AC-induced Sphk1 activity through Gi. DOP and pFTY720 act in an inhibitory manner on rolling by downregulating S1P1 and S1P3, but in addition, pFTY720 directly inhibits the Gq-dependent part of S1P-incuded S1P3-dependent P-selectin mobilization. In summary, the pro-rolling effect of S1P3 dominates over the anti-rolling of S1P_1_, and the net effect of acute S1P exposure is an overall increase in P-selectin-dependent leukocyte rolling. AC, adenylate cyclase; AUY 954, S1P1-specific agonist; β2AR, β2 adrenergic receptor; DOP, the S1P lyase inhibitor 4-deoxypyridoxine; H1, H1 histamine receptor; PSGL, P-selectin glycoprotein ligand-1; Sphk1, sphingosine kinase 1; TY-52156, S1P_3_-specific inhibitor.

Our study reveals the following six important and novel findings (Fig. 8): (1) we provide evidence that S1P is a direct agonist of P-selectin mobilization and leukocyte rolling; (2) we identify S1P_3_ as the major responsible S1P receptor; (3) we show that several P-selectin agonists employ S1P and S1P_3_ as mediators of their P-selectin-mobilizing effects by activating Sphk1; (4) we characterize a novel cAMP-dependent mechanism of Sphk1 activation and P-selectin mobilization; (5) we identify a rolling-inhibitory effect of endothelial S1P_1_; and (6) we show that pharmacological inhibition or downregulation of S1P_3_ for example by FTY720 attenuates leukocyte rolling in a physiological setting. Only few studies have addressed S1P in the context of processes related to leukocyte rolling: (1) S1P has been demonstrated to both induce and inhibit the exocytosis of von Willebrand factor through different pathways^49^; (2) Sphk1 was suggested to play no role in basal leukocyte rolling but to promote rolling after exogenous histamine administration^16^; (3) although S1P promoted firm neutrophil adhesion *in vitro*^50^, neutrophil recruitment was inhibited during acute inflammatory lung injury in S1P lyase-deficient mice^51^, with S1P lyase inhibitors^52^ and by S1P administration^53^, respectively, and (4) FTY720 was shown to suppress neutrophil recruitment in several disease models^3^,^53^,^54^. This has made general conclusions on the role of S1P and its receptors in leukocyte rolling or their mode of action rather difficult.

Our findings add some new evidence that helps to better understand the role of S1P in rolling. First, it is crucial to distinguish acute S1P effects on pre-stored P-selectin occurring within minutes from S1P effects on cytokine-induced P-selectin transcription that takes hours. Second, the acute S1P effects on P-selectin are different from those caused by continuous exposure to high S1P levels or the lack of S1P receptors. Third, the effects of S1P analogues such as FTY720 may differ considerably from the effects of genuine S1P at the receptor level^55^. In respect to rapid P-selectin mobilization, we have clearly shown that S1P is a direct P-selectin agonist and leads to increased P-selectin-dependent leukocyte rolling within minutes of its direct administration into the circulation. It is known that despite the high amounts of S1P already present in the blood, intravascular injection of S1P can still cause functional effects on the endothelium such as attenuation of vascular leakage, vasodilatation and protection against reperfusion injury^53^,^56^,^57^. Accordingly, the reduced leukocyte rolling we observed after pharmacological S1P_3_ inhibition and in *S1P_3_*^*−/−*^ mice, respectively, are strong arguments for the causal and direct positive contribution of endothelial S1P signalling to P-selectin-dependent leukocyte rolling. However, we have also found evidence that endothelial S1P_1_ plays an opposite role in rolling as its deletion led to a rolling increase. However, the pro-rolling effect of S1P_3_ appears to be dominant over the anti-rolling effect of S1P_1_ as the net effect of acute intravascular S1P administration in wild-type mice (where both receptors are being engaged) was an overall increase in rolling. However, in the absence of S1P_3_, anti-rolling effects of S1P_1_ may also contribute to the reduction in rolling. In fact, there was a minor but significant increase in rolling with FTY720 in S1P_3_ deficiency that may have occurred because of S1P_1_ downregulation by FTY720. Accordingly, in the absence of endothelial S1P_1_, the increase in rolling may be due to a combination of the loss of anti-rolling S1P_1_ effects and the still intact and potent pro-rolling S1P_3_ effects. Indeed, in *S1P*_*1*_^*−/−*^ mice, FTY720 inhibited rolling to lower than basal levels.

We have identified two possible sources of endogenous S1P involved in P-selectin mobilization in our model: interstitial mast cells and endothelial cells (Fig. 8). Interstitial mast cell activation following mechanical cremaster muscle manipulation is entirely responsible for the leukocyte rolling occurring there, and histamine release accounts for ~50% of it (ref. 29). As S1P can be released by allergically activated mast cells^45^, and physical stimuli such as pressure and heat can activate mast cells^46^,^47^, we hypothesized that S1P may be another mast cell product contributing to the remaining part of leukocyte rolling. The physical stimulus we employed to test this in mast cells was a short, harmless temperature elevation^46^,^47^. Indeed, we found them to release S1P within minutes that mobilized P-selectin in HUVEC in part in a S1P_3_-dependent manner. Whether this occurs *in vivo* and actually contributes to leukocyte rolling in our model should be addressed by future studies. Although platelets are the major source of P-selectin, elaborate studies have shown that platelet P-selectin does not play a role in leukocyte rolling^58^,^59^, and, in our hands, S1P had no effect on basal or thrombin-stimulated P-selectin in platelets; neither was platelet activation altered in *Sphk1*^−/−^ mice (Supplementary Fig. 9).

We have identified endothelial cells as the second source of endogenous S1P capable of inducing P-Selectin mobilization. Sphk1 activation has been known to occur after stimulation with inflammatory cytokines as well as histamine; however, the pathway by which it contributes to rolling has remained unclear^16^. We provide evidence that S1P produced by histamine-activated Sphk1 contributes to full-blown histamine mobilization of P-selectin through S1P_3_. The evidence stems from two observations: (1) the partial inhibition of P-selectin mobilization after histamine by the blockade of Sphk1 or S1P_3_ and (2) the elimination of the inhibitory effect of Sphk1 blockade on P-selectin by exogenous S1P. Coincidently, the S1P_3_ receptor, the H1 histamine receptor and the thrombin PAR-1 receptor^41^ not only all mobilize P-selectin but also couple to G_q_; on the other side, pharmacological G_q_ inhibition has been shown to inhibit P-selectin exocytosis at least in platelets^60^. This offers a plausible explanation of why S1P_3_ effects have gone unnoticed in experiments employing H1 blockers or G_q_ inhibitors. Our finding that endothelial-specific *G*_*q/11*_*−/−* mice have reduced P-selectin-dependent leukocyte rolling can certainly be explained by the blockade of several G_q_-coupled receptors including S1P_3_. Indeed, all these agonists have their own S1P-independent pathways of inducing P-selectin as neither S1P_3_ nor Sphk1 deletion/inhibition was able to completely inhibit P-selectin mobilization. However, part of the histamine effect can be attributed to S1P_3_. Our observation that rolling could not be reduced any further by pharmacological S1P_3_ inhibition in G_q/11_−/− mice argues that G_q_ mediates the pro-rolling effect of S1P_3_. The site of action of S1P released by interstitial mast cells or by endothelial cells exposed to histamine from the same mast cells would primarily be on the abluminal side of the vascular endothelium. This complies with an oriented signalling mode as the abluminal side should be reached first by any locally released interstitial agents. This scenario is in line with the dynamic signalling model proposed by Camerer *et al*.^7^ of how abluminal S1P signalling maintains endothelial integrity despite high S1P levels in blood.

We were also intrigued by the observation that not only histamine but another P-selectin-mobilizing agonist such as epinephrine^41^ that couples with G_s_ and not G_q_ employed Sphk1 and S1P_3_ for full-scale P-selectin mobilization (Fig. 8). We have demonstrated that epinephrine also activated Sphk1 but through a cAMP-dependent mechanism and — similar to histamine — also employed S1P and S1P_3_ to help mobilize P-selectin. Indeed, direct stimulation of Sphk1 by forskolin (as measured by the conversion of C17-sphingosine to C17-S1P) mobilized P-selectin through S1P_3_ *in vitro* and induced leukocyte rolling *in vivo*. In agreement with a role for cAMP, forskolin has been shown to activate Sphk1 in pheochromocytoma cells years ago^42^. However, S1P_3_ or Sphk1 inhibition did not completely abrogate the effects of epinephrine or forskolin, suggesting that there must be additional pathways of how cAMP mobilizes P-selectin^41^. Interestingly, the pathway of cAMP-stimulated Sphk1 provides a putative mechanism of how S1P_1_, with its established inhibitory effect on cAMP generation^4^, may be exerting its antirolling effect. Indeed, the highly specific S1P_1_ agonist AUI 954 (ref. 48) inhibited C17-S1P generation after forskolin along with lowering cAMP (Supplementary Fig. 8). This suggests that S1P_1_ may be attenuating cAMP-induced Sphk1 activation in a negative-feedback manner, thereby restricting the amounts of S1P available for P-selectin mobilization by S1P_3_. The reduced leukocyte rolling we have observed in *Sphk1*^*−/−*^ mice is in line with Sphk1/S1P serving as a general transmission/amplification hub for different incoming P-selectin mobilization signals. While the S1P_1_ receptor has often been implicated in Sphk1 signalling, there is to our knowledge only scarce evidence for an involvement of S1P_3_ in conveying Sphk1 signals: only one study has implied S1P_3_ as a mediator of thrombin-induced Sphk1 effects in dendritic cells^61^. The requirement of S1P_3_ for full efficiency of several P-selectin agonists may also serve another purpose: S1P_3_ downregulation or desensitization after engagement by endogenous S1P may help ameliorate excessive P-selectin stimulation and help turn it off.

Acute S1P effects on P-selectin mobilization should be distinguished from those caused by continuous exposure to S1P or S1P analogues. High S1P levels after S1P lyase inhibition or deficiency and administration of FTY720 have been shown to downregulate S1P receptors^1^,^62^,^63^ including S1P_3_ (ref. 64) and impair lymphocyte trafficking. Accordingly, the reduced neutrophil recruitment found in S1P lyase-deficient mice^51^ or after FTY720 treatment^53^,^54^ may be due to downregulation of S1P_3_. In line with this, we have observed that FTY720 had no additional inhibitory effect on rolling in *S1P*_*3*_^*−/−*^ mice. However, another mechanism may also be involved: we have previously shown that pFTY720 is a potent inhibitor of G_q_-mediated S1P-induced S1P_3_ signalling^39^. In the present study, we extended this observation to P-selectin mobilization by S1P_3_. Thus, both mechanisms could be taking place simultaneously as: (1) preincubation with pFTY720 abolished S1P-induced P-selectin mobilization in endothelial cells as well as S1P_3_-dependent S1P signalling in S1P_3_-CHO cells; (2) pFTY720 had a minor and very short-lived positive effect on P-selectin compared with S1P, while (3) its simultaneous application together with S1P abolished P-selectin mobilization by S1P. In line with this interpretation, another P-selectin-dependent process, the recruitment of early thymic progenitors to the thymic endothelium, has been shown to be inhibited by FTY720 (ref. 65), although the responsible receptor was not identified.

In summary, our study suggests an important role for S1P and S1P_3_ in the induction of rapid endothelial P-selectin mobilization and consecutive P-selectin-dependent leukocyte rolling. S1P acts thereby both directly as an agonist and, indirectly, as a contributor to P-selectin mobilization by other P-selectin agonists.

## Methods

### Mice

*S1P*_*3*_^*−/−*^ mice were provided by Jerold Chun, Scripps Research Institute to B.L. and crossbred to *C57Bl6J* for more than six generations. *Sphk1*^*−/−*^ mice were provided to M.H.G. by Richard Proia (NIH) and Tie2-Cre-ER(T2); *Gα*_*q*_^*flox/flox*^*;Gα*_*11*_*−/−* were from S.O. Mice deficient for the endothelial S1P_1_ receptor were generated by crossbreeding *S1P*_*1*_^*flox/flox*^ mice^66^ with *endothelial-SCL-Cre-ER*^*T*^ mice, in which the tamoxifen-inducible Cre-ER^T^ recombinase is driven by the 5′-endothelial enhancer of the stem cell leukaemia (SCL) locus^67^ (Supplementary Fig. 11). Cre-negative *S1P*_*1*_^*SCL-Cre-ERT*^ littermates treated with the same tamoxifen regimen were used as controls. Recombination was induced by daily intraperitoneal injections of tamoxifen (40 mg per kg body weight) for five consecutive days, and the experiments were performed 6 weeks later. Successful recombination was shown in lung endothelial cells isolated using CD31-coated magnetic beads (Dynabeads) by site-specific PCR for the excised exon. All mice used in the study were male and at least 10 weeks of age. All procedures were performed in accordance with the institutional guidelines for health and care of experimental animals and were approved by the Regierung von Baden Württemberg and Oberbayern, respectively.

### Substances and antibodies

S1P was purchased from Enzo Life Science. The S1P_3_ inhibitors TY-52156 (Supplementary Fig. 2) and FTY720 were synthesized as described^32^. AUY 954 was a kind gift from Klaus Seuwen (Novartis). *In vivo*, FTY720 and TY-52156 were applied intraperitoneally at 1.25 mg per kg and forskolin at 1.3 mg per kg body weight 30 min or 24 h before the experiment as indicated. S1P was injected via a carotid artery catheter at 30 μg per kg body weight (125 μM S1P solution in 2% BSA per 0.9% NaCl). The S1P lyase inhibitor DOP (Sigma Aldrich) was supplemented in the drinking water (30 mg l^−1^) for 1 week. W146 and C17 sphingosine were from Avanti polar lipids, the SKi 4-[[4-(4-chlorophenyl)-2-thiazolyl]amino]-phenol (SKi, SKI II and SPHK I2), pFTY720, SEW2871 and U73122 from Cayman Chemical Co; epinephrine from Sanofi Aventis; forskolin from Calbiochem; IBMX, cpt-cAMP, diphenhydramine and cimetidine from Sigma Aldrich; and thapsigargin from AppliChem. S1P_1_-CHO and S1P_3_-CHO cells were from Novartis. The following antibodies were used: CD62P-PE (phycoerythrin) anti-mouse (eBioscience, 12-0626-82, 3:100), CD4-PE anti-mouse (eBioscience, 12-0041, 1:100), CD8-PE anti-mouse (eBioscience, 12-0081, 1.4:100), Ter119-PE anti-mouse (Miltenyi, 130-091-783, 3:100), Ly6G&Ly6C-PE anti-mouse (BD Pharmingen, 553128, 1.6:100), CD45R-PE anti-mouse (BD Pharmingen, 553089, 1.6:100), CD34-AlexaFluor700 anti-mouse (BD Pharmingen, 1.6:100), CD16/32-PerCpCy5.5 anti-mouse (BD Pharmingen, 560540, 1.65:100), LyA/E-FITC anti-mouse (BD Pharmingen, 557405, 1.1:100), CD117-PE-Cy7 anti-mouse (BD Pharmingen, 558163, 1.7:100), CD450-V450 anti-mouse (BD Pharmingen, 560697, 1.8:100) and P-selectin glycoprotein ligand-1-PE anti-mouse (BD Pharmingen, 555306, 1:1,000). The P-selectin-blocking rat-anti-mouse monoclonal antibody RB40.34 was a generous gift from Dietmar Vestweber (MPI Münster).

### Flow chamber assay for rolling on immobilized P-selectin

Rectangular glass capillaries (0.4 × 0.04 mm, VitroCom) were used for *ex vivo* flow chamber experiments as described^31^. Chambers were coated with recombinant murine (rm)P-selectin (CD62-P, 20 μg ml^−l^, R&D Systems). Following overnight incubation at 4 °C, flow chambers were blocked with 5% casein (Sigma-Aldrich) in PBS for 2 h. For experiments, one end of the chamber was attached to a carotid artery catheter, while the other was left open to regulate blood flow through the microflow chamber. Microscopy was performed using a modified Olympus BX51 upright microscope (LaVision Biotec) with a saline immersion objective (40/0.8 numerical aperture (NA), Olympus). Leukocyte rolling was recorded over 10 min per microflow chamber with a CCD (charge-coupled device) video camera (model CF8/1; Kappa) connected to a Panasonic S-VHS recorder and analysed offline.

### Intravital microscopy of the cremaster muscle

Intravital microscopy of postcapillary venules of the mouse cremaster muscle was used to study leukocyte rolling under different inflammatory conditions^25^,^27^. Briefly, mice were anaesthetized by intraperitoneal injection of ketamine and xylazine (100 and 20 mg per kg body weight, respectively), after which a tracheal tube was inserted and the carotid artery cannulated for blood sampling and systemic application of substances. Thereafter, the scrotum was opened, the cremaster muscle exteriorized, spread over a cover glass and superfused with warmed (35 °C) bicarbonate-buffered saline. Observation of postcapillary venules was conducted on an upright microscope (Olympus BX51) with a saline immersion objective ( × 40/0.8 NA). To abolish P-selectin-dependent rolling, the P-selectin-blocking monoclonal antibody RB40.34 (30 μg per mouse) was diluted in 200 μl saline and injected via the carotid artery catheter. Experiments were recorded via a CCD camera system (model CF8/1; Kappa) on a Panasonic S-VHS recorder. Systemic blood samples (10 μl) were obtained before and during the experiment, stained using Turck’s solution and assessed for white blood cell count using a haemocytometer. Data analysis was performed offline using the video tapes from *in vivo* experiments. Diameter, segment length of postcapillary venules and venular centreline blood flow velocity were assessed using a digital image-processing system and a digital online cross-correlation programme, respectively (Circusoft Instrumentation)^24^. Leukocyte rolling flux fraction was calculated from the number of rolling cells that crossed a perpendicular line through a given vessel within 1 min in relation to the total number of circulating leukocytes.

### Whole-mount immunohistochemistry

For immunohistochemical detection of luminal endothelial P-selectin expression in cremaster muscle whole mounts, primary monoclonal antibody against P-selectin RB40.34 (30 μg per mouse) was systemically injected after 20-min superfusion of the exteriorized right cremaster muscle ensuring binding to P-selectin on the luminal vessel surface. To remove excess antibody, the inferior vena cava was cut and animals were perfused with 10 ml of 0.9% NaCl. The left cremaster muscle was exteriorized *post mortem* to serve as unstimulated negative control. The cremaster muscles were then mounted on adhesive slides (Superfrost), transferred into −20° acetone over night and stored at −80 °C until further processing. For staining of P-selectin expression, cremaster whole mounts were permeabilized with saponin (0.03% in Tris-buffer) and incubated with 2 μg ml^−l^ biotinylated secondary goat anti-rat IgG (Southern Biotechnology Associates Inc, Cat. No 3030-08). Detection of antigen–antibody complexes was carried out with a commercial biotinylated horseradish peroxidase/avidin complex (Vectastain ABC, Vector Laboratories) according to the manufacturer’s instructions. Slides were counterstained using Mayer’s Hemalaun and analysed using a Zeiss microscope with a × 100, 1.4 NA oil immersion objective.

### P-selectin mobilization in HUVEC

HUVECs (passage 1–4) were cultured in 20% human serum/RPMI. After incubation in serum-free medium for 4 h, cells were stimulated with histamine (0.25 or 25 μM), S1P (1 μM), SEW2871 (1 μM) and epinephrine (9 μM)/IBMX (100 nM), respectively, for the indicated times. Before histamine or S1P stimulation, the S1P_3_ inhibitor TY-52156, SKi or phosphorylated FTY720 (10 μM) were added for 30 min; with forskolin, inhibitors were added 10 min before stimulation. Five minutes before the end of the experiment, PE-conjugated anti-P-selectin antibody (eBioscience; 1.6:100) was added directly into the 500 μl of incubation medium after which the cells were washed twice and analysed immediately by flow cytometry.

### Stimulation with supernatants of activated mast cells

Mouse peritoneal mast cells were isolated by a Percoll gradient as described^68^ and characterized as CD45^+^, Lin^−^ (CD45R, Ly6G, Ly6C, Ter119, CD4 and CD8), CD117^+^, Sca-1^+^, CD16/32^+^ and CD34^+^ cells by flow cytometry and consecutive toluidine blue staining^68^,^69^. The yield was 0.05–0.1 × 10^6^ mast cells per mouse. All peritoneal cells were incubated with 10 μM sphingosine for 30 min at 37 °C before the Percoll gradient isolation procedure. Stimulation was performed by adding 250 μl warm (53 °C) 2% BSA/alphaMEM to 0.5 × 10^6^ mast cells and subsequent incubation for 2 min at 53 °C as described^46^,^47^. For stimulation of HUVEC, 100 μl of the mast cell supernatant was supplemented with diphenhydramine and cimetidine (10 μM each) and added to confluent HUVEC cultured in 200 μl RPMI for 10 min. PE-conjugated anti-P-selectin antibody was added directly into the 300 μl at the time of stimulation. Cells were washed twice with serum-free medium, trypsinized and analysed by flow cytometry.

### Measurements of Sphk1 activity using C17-sphingosine

Five μM C17-sphingosine was added together with 10 μM forskolin to HUVEC in RPMI with 2 mg per ml BSA fraction V (Serva) for 30 min. Cells were washed extensively and lysed in 50 mM Tris/HCl (pH 7.5), 0.5%NP-40, 10% glycerol, 250 mM NaCl, 5 mM EDTA, 0.5 mM phenylmethyl sulphonyl fluoride, 5 μg ml^−l^ leupeptin and aprotinin. Protein concentration was measured using the Pierce BCA Protein Assay Kit (Thermo Scientific). C17-S1P was determined by mass spectrometry as described^70^ and expressed per milligram cell protein.

### Statistics

For pair-wise comparison between experimental groups, a Wilcoxon rank sum test or paired Student’s *t*-test was performed. For multiple comparisons, a Kruskal–Wallis nonparametric analysis of variance on ranks was performed followed by Dunn’s *post hoc* test. A *P* value<0.05 was considered statistically significant. All statistical analyses were carried out with GraphPad Prism 5.2 (PraphPad Software Inc, La Jolla). Data are presented as mean±s.e.m. Sample size has been provided for each experiment in the figure legend (no exclusion criteria, randomization or blinding).

## Author contributions

C.N., S.B., C.F.G.-d.-A., P.K., V.P.P., I.R., G.H., K.v.W.L. and H.K. performed research, collected data, analysed and interpreted data, performed statistical analysis, wrote the manuscript. M.H.G., H.C.d.C.F.N., J.G., G.H., B.L.-S. and S.O. contributed vital reagents or analytical tools and interpreted data. M.S. and B.L. designed research, analysed and interpreted data, and wrote the manuscript.

## Additional information

**How to cite this article:** Nussbaum, C. *et al.* Sphingosine-1-phosphate receptor 3 promotes leukocyte rolling by mobilizing endothelial P-selectin. *Nat. Commun.* 6:6416 doi: 10.1038/ncomms7416 (2015).

## Supplementary Material

Supplementary Information Supplementary Figures 1-11, Supplementary Table 1 and Supplementary References

## Figures and Tables

**Table 1:** Haemodynamic parameters of mouse cremaster muscle experiments.

**Mouse strain**	**Treatment**	**Fig.**	**Mice (*****n*****)**	**Venules**	**Diameter (μm)**	**Centreline velocity (μm s**^−1^**)**	**Shear rate (s**^−1^**)**	**WBC ( × 10**^3 ^**μl**^−1^**)**
*C57Bl6*	*None*	1a	6	21	30.0±1.1	1,876±215	1,562±160	5,704±622
*S1P*_*3*_^*−/−*^			5	20	28.8±1.4	1,885±182	1,449±203	8,060±983
*C57Bl6*	*None*	1c	5	16	33.6±1.6	2,181±324	1,693±319	6,659±192
*C57B6*	*S1P*_*3*_*i*		4	17	29.9±1.0	1,861±183	1,614±138	5,808±633
*C57Bl6*	*None*	1d	5	5	34.9±1.6	1,638±406	1,150±270	5,032±613
	*S1P*		5	5	34.0±1.8	1,427±408	1,006±248	4,144±367
*C57Bl6*	*None*	2c	4	17	29.3±1.9	2,635±195	2,303±188	5,633±254
*G*_*q/11*_^*−/−*^	*None*		5	22	30.8±1.3	2,973±162	2,445±160	6,405±163
*G*_*q/11*_^*−/−*^	*S1P*_*3*_*i*		4	22	29.7±1.2	1,623±113*	1,367±93*	3,348±1,076
*C57Bl6*	*None*	3a	5	26	33.4±1.1	2,017±163	1,524±131	7,043±899
	*FTY 30 min*		6	36	34.3±0.8	1,857±127	1,380±94	4,270±203
	*FTY 24 h*		6	35	32.5±1.0	1,540±100	1,178±71	1,110±152*
*C57Bl6*	*None*†	3b	6	21	30.0±1.1	1,876±215	1,562±160	5,704±622
	*FTY 30 min*		8	32	29.4±0.6	2,419±203	2,033±175	7,147±343
*S1P*_*3*_^*−/−*^	*None*†		5	20	28.8±1.4	1,885±182	1,449±203	8,060±983
	*FTY 30 min*		5	19	29.2±0.6	2,384±196	2,014±185*	6,998±937
*C57Bl6*	*Normal diet*	3c	4	17	30.3±0.7	1,894±185	1,510±132	4,819±1,090
	*DOP diet*		5	23	30.3±0.9	1,839±186	1,476±125	2,415±297
*C57Bl6*	*None*	4c	4	16	33.6±1.0	1,613±145	1,178±101	8,143±548
*Sphk1*^*−/−*^			4	15	33.7±1.7	1,709±217	1,420±241	7,760±299
*C57Bl6*	*None*‡	5e	5	26	33.4±1.1	2,017±163	1,524±131	7,043±899
	*Forskolin*		5	21	30.4±1.2	2,143±163	1,768±147	3,382±579*
*S1P*_*1*_^*SCL-Cre-ERT*^ *Cre^−^*	*None*	7a	9	32	30.1±0.8	1,778±153	1,465±127	3,250±567
*S1P*_*1*_^*SCL-Cre-ERT*^ *Cre^+^*			9	35	29.1±0.6	1,849±172	1,546±136	2,238±289
*S1P*_*1*_^*SCL-Cre-ERT*^ *Cre^−^*	*None*§	7b	9	32	30.1±0.8	1,778±153	1,465±127	3,250±567
	*FTY720*		3	12	27.7±0.9	1,675±209	1,488±184	2,280±742
*S1P*_*1*_^*SCL-Cre-ERT*^ *Cre^+^*	*None*§		9	35	29.1±0.6	1,849±172	1,546±136	2,238±289
	*FTY720*		4	16	27.8±0.9	2,237±232	1,999±213	3,015±459

DOP, 4-deoxypyridoxine; S1P, sphingosine-1-phosphate; SCL, stem cell leukaemia; WBC, white blood cell.

^*^P<0.05 versus respective control (‘none’).

^†^Data are identical to Fig. 1a (‘none’).

^‡^Data are identical to Fig. 3a (‘none’).

^§^Data are identical to Fig. 7a (‘none’).
