# Supplementary material for: Sphingosine-1-phosphate receptor 3 promotes leukocyte rolling by mobilizing endothelial P-selectin
Source: Nat Commun. 2015 Apr 2;6:6416. doi: 10.1038/ncomms7416 (PMC4396399; doi:10.1038/ncomms7416)
Supplement: Supplementary Information — Supplementary Figures 1-11, Supplementary Table 1 and Supplementary References [file ncomms7416-s1.pdf]

Supplementary Figure 1: No difference in leukocyte rolling velocities between *C57Bl6* mice, *S1P$_3$$^{-/-}$*, *S1P$_1$$^{SCL\text{-}Cre\text{-}ERT}$* mice and their respective controls as well as FTY720 and DOP-treated mice. Data are presented as mean±s.e.m. For statistical analysis a Wilcoxon rank sum test was performed.

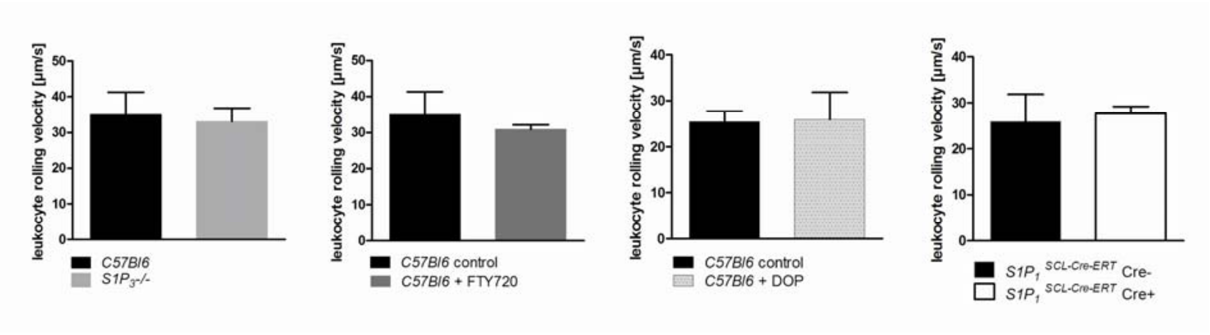

Supplementary Figure 2: Synthesis of the S1P$_3$ inhibitor TY-52156. Pinacolone was reacted with ethylacetate and potassium *tert*-butoxide in diisopropyl ether to give 5,5-dimethylhexane-2,4-dione with 42 % yield according to (1). This diketone was chlorinated at alpha-position following the literature (2) using chlorotrimethylsilane and dimethyl sulfoxide in dry acetonitrile in presence of a catalytic amount of the phase transfer catalyst TBAB to give 62% 3-chloro-5,5-dimethylhexane-2,4-dione after column chromatography. In the next step this compound was coupled with *p*-chlorophenyldiazonium chloride to provide (*E*)-*N*'-(4-chloro-phenyl)-3,3-dimethyl-2-oxobutanehydrazonoyl chloride in 32 % yield as a yellow solid. Finally this hydrazonoyl chloride was reacted with 4-chloroaniline in ethanol in the presence of triethyl-amine to give 81% of the desired (*E*)-*N,N'*-bis(4-chlorophenyl)-3,3-dimethyl-2-oxobutane-hydrazonamide (TY-52156) as an yellow powder.

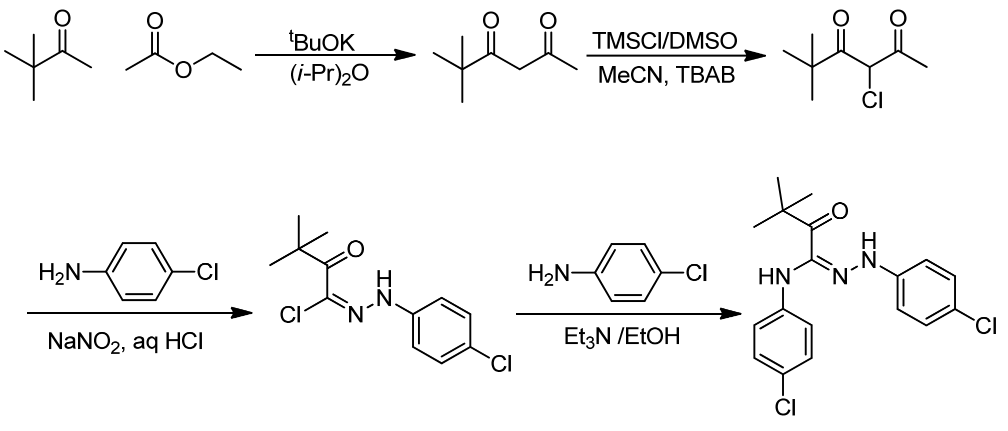

Supplementary Figure 3: Specificity of the $S1P_3$ inhibitor TY-52156 for $S1P_3$ as shown in $S1P_1$- and $S1P_3$-overexpressing CHO cells. Cells were preincubated with or without 10µM TYP-52156 for 30 min and stimulated with 1µM S1P for 5 and 10 min. Western blotting for phospho Erks (pp44/42 MAPK) was performed on total cell lysates. Of note, the Erk response in $S1P_3$-CHO lasted longer than in $S1P_1$-CHO cells.

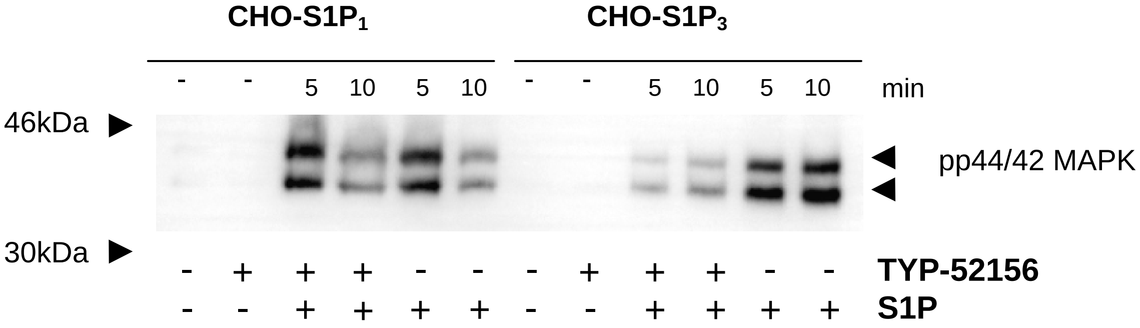

Supplementary Figure 4: P-selectin mobilization in HUVEC. (Left) Representative flow cytometry histogram for P-Selectin mobilization in HUVEC after 5 min of 25 µM histamine stimulation. (Right) HUVEC were stimulated with 0.25 µM histamine in the presence or absence of the H1 receptor blocker diphenhydramine (10 µM). P-selectin was analysed by flow cytometry (fold of control). Quantitative data are presented as mean±s.e.m for at least 3 independent experiments. Significance was established using a paired two-tailed Student's *t*-test. * $P < 0.05$.

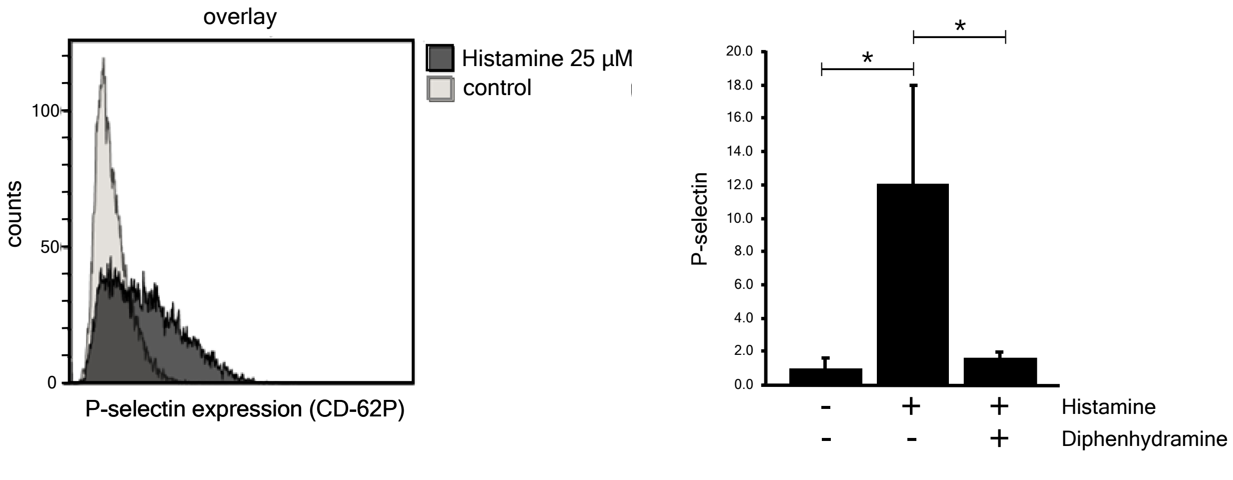

Supplementary Figure 5: Unaltered rolling of neutrophils from DOP-treated mice on immobilized P-selectin. The number of rolling cells in whole blood was quantified in microflow chambers coated with recombinant murine P-selectin and perfused with arterial blood diverted from the carotid artery of control and DOP-treated mice (n=3 each) for the indicated times. FOV (field of view). Quantitative data are presented as mean±s.e.m. For statistical analysis a Wilcoxon rank sum test was performed.

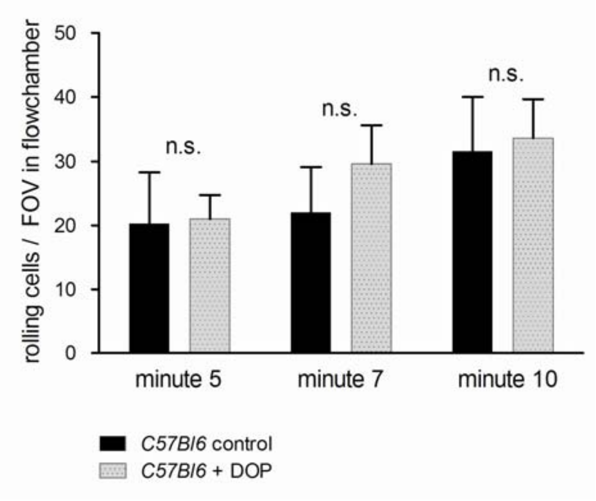

Supplementary Figure 6: Pre-incubation with pFTY720 or S1P desensitizes CHO-S1P$_3$ cells to consecutive S1P stimulation. CHO-S1P$_3$ were incubated with or without 1 μM pFTY720 or 1 μM S1P for 30 min and washed extensively. Fresh medium was added for another 30 min, after which cells were stimulated with 1 μM S1P. For comparison, cells without any pre-incubation were stimulated 1 μM pFTY720. Erk (p44/42 MAPK) phosphorylation was detected by Western blotting. In red is shown the original S1P response, in dark blue the S1P response after pre-incubation with pFTY720, and in light blue the S1P response after pre-incubation with S1P.

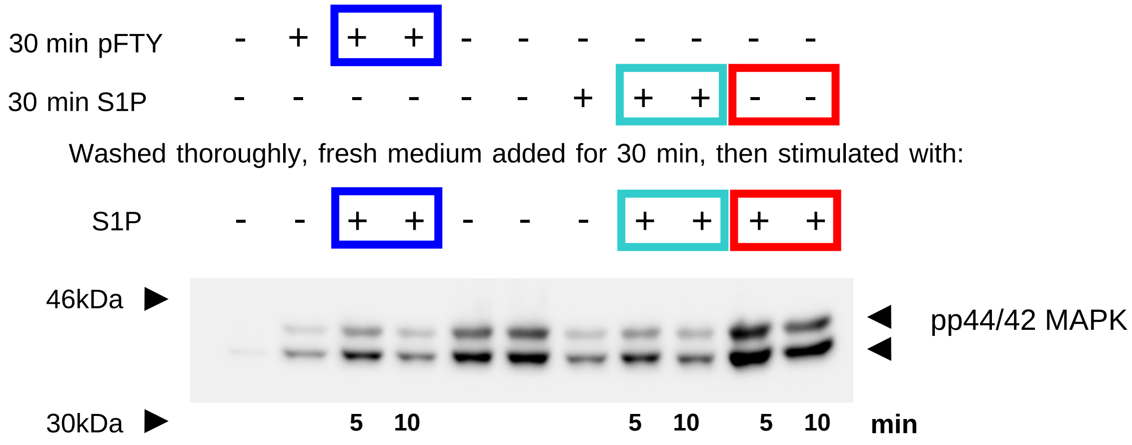

Supplementary Figure 7: Biological proof of the presence of biologically active S1P in mast cell supernatants after physical stimulation. CHO cells overexpressing $S1P_1$ were stimulated with supernatants from native mast cells and mast cells stimulated as detailed in the manuscript, respectively, in the presence or absence of 10 μM W146 ($S1P_1$-receptor antagonist) for 5 min. Erk phosphorylation was detected by Western blotting. The supernatant of stimulated mast cells contained ~10 nM mast cell-derived S1P as determined by mass spectrometry.

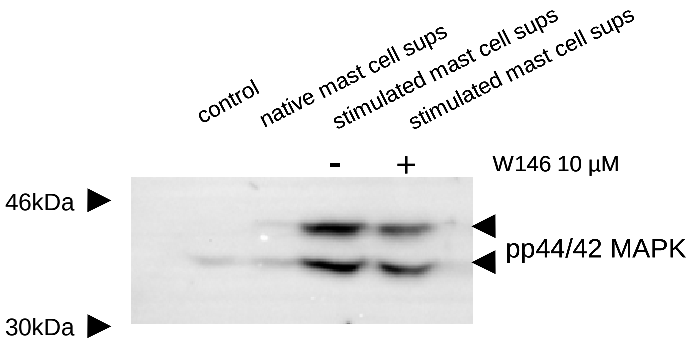

Supplementary Figure 8: C17-S1P and cAMP synthesis are induced by forskolin and blocked by the $S1P_1$ agonist AUY 954. (**a**) HUVEC were stimulated with forskolin (10 μM) for 30 min in the presence or absence of 1 μM AUY 954 (added 1 min before forskolin). Cells were harvested and C17-S1P synthesized from C17-sphingosine was measured by mass spectrometry as described in the manuscript. (**b**) HUVEC were stimulated with forskolin (10 μM) in the presence or absence of 1 μM AUY 954 or 1 μM S1P for 30 min. Intracellular cAMP per 10^6 cells was measured by Amersham cAMP Biotrak EIA System (GE Healthcare). Quantitative data are presented as mean±s.e.m. from at least 3 independent experiments. Significance was established using a paired two-tailed Student's *t*-test. * $P < 0.05$

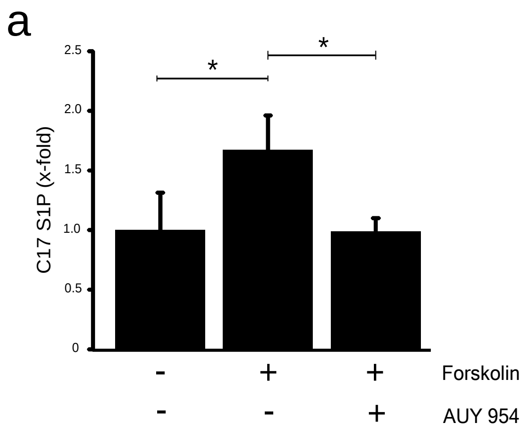

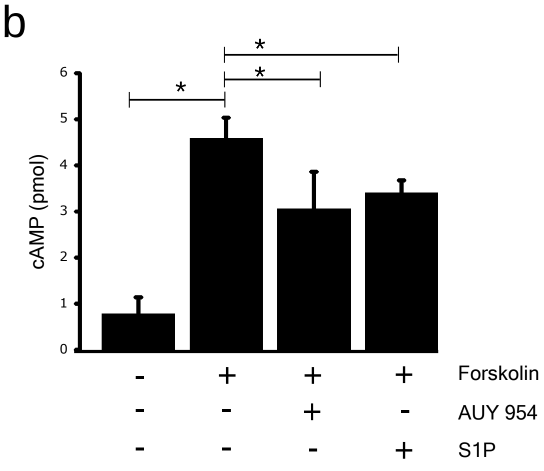

Supplementary Figure 9: Lack of P-selectin mobilization by S1P in platelets. (**a**) Isolated mouse platelets were stimulated with 1 µM S1P, 0.1 U thrombin or both for 5 min, and P-selectin was analyzed by flow cytometry. (**b**) Basal P-selectin expression and thrombin-induced P-selectin mobilization (0.1 U thrombin for 5 min) were analyzed by flow cytometry in isolated platelets of *C57Bl6* and *SphK1-/-* mice. (**c**) qRT-real time PCR analysis of S1P receptor mRNA expression in isolated mouse platelets. Data are expressed as fold of the mRNA level of $S1P_1$. Quantitative data are presented as mean±s.e.m from at least 3 independent experiments. Significance was established using a paired two-tailed Student's *t*-test. * $P < 0.05$.

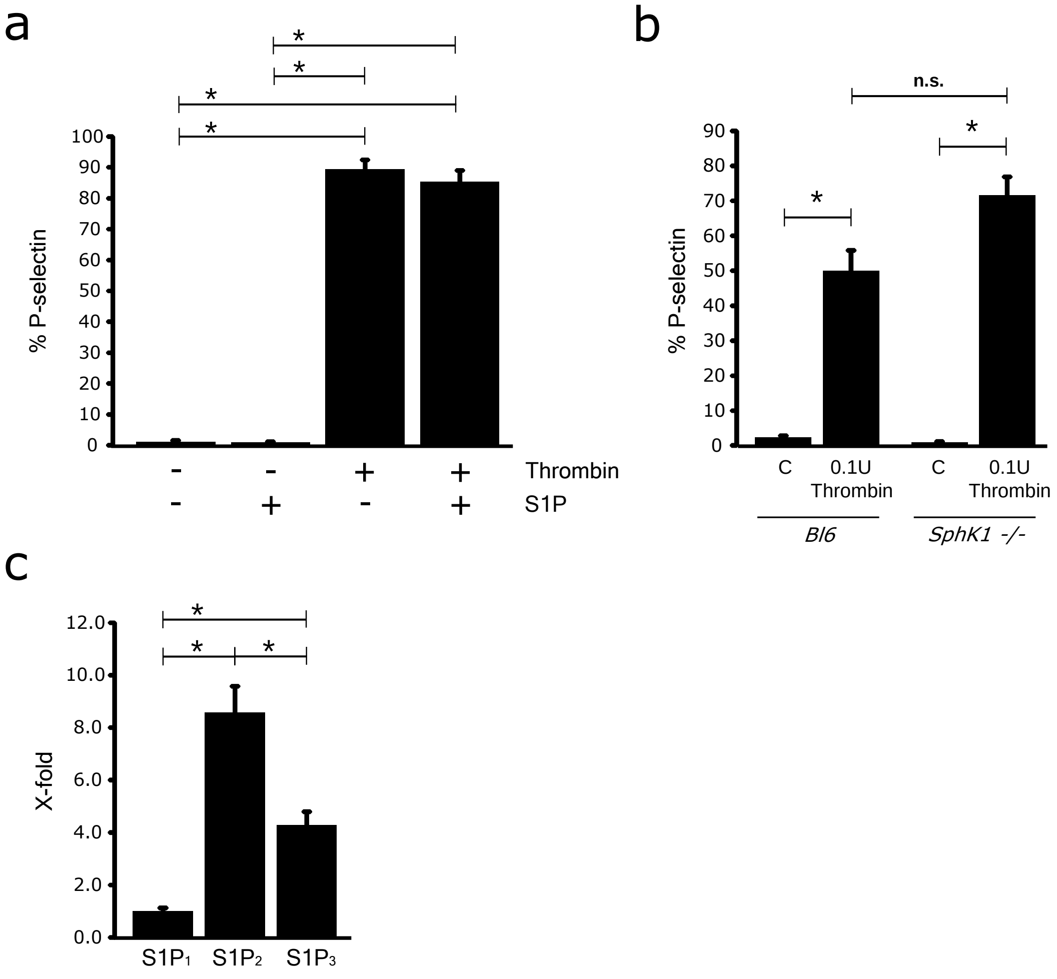

Gene	Forward (5´-3´)	Reverse (3´-5´)
S1P$_1$	ATGGTGTCCACTAGCATCCC	CGATGTTCAACTTGCCTGTGTAG
S1P$_2$	ATGGGCGGCTTATACTCAGAG	GCGCAGCACAAGATGATGAT
S1P$_3$	ACTCTCCGGGAACATTACGAT	CAAGACGATGAAGCTACAGGTG

Supplementary Figure 10: The $S1P_3$ inhibitor TY-52156 inhibits S1P-induced P-selectin mobilization in a concentration-dependent manner. HUVEC were stimulated with 1 µM S1P for 5 min in the presence or absence of 1 µM and 10 µM TY-52156. Quantitative data are presented as mean±s.e.m. from at least 5 independent experiments each. Significance was established using a paired two-tailed Student's *t*-test. * $P < 0.05$

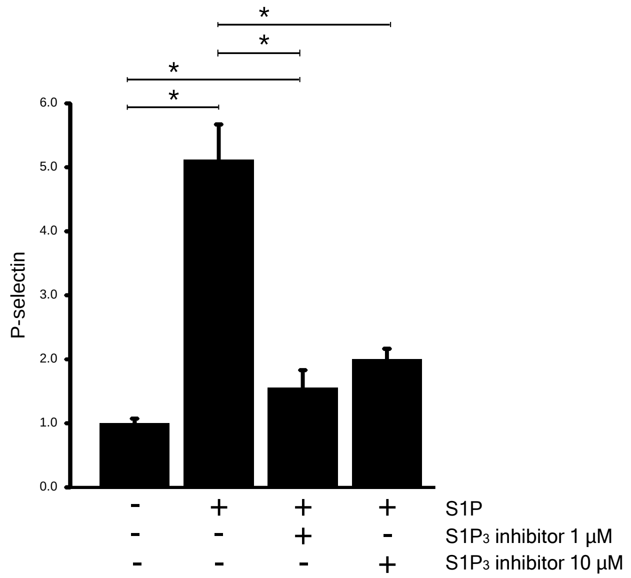

Supplementary Figure 11: Proof of successful deletion of *$S1P_1$* in *$S1P_1^{SCL\text{-}Cre\text{-}ERT}$* mice. Representative PCR for the deleted *$S1P_1$* allele using excision-specific primers in endothelial cells enriched from digested lungs of *$S1P_1^{SCL\text{-}Cre\text{-}ERT}$* mice sacrificed 6 weeks after a 5 day-course of tamoxifen treatment (4 *$S1P_1^{flox/flox}$* mice transgenic for *SCL-Cre-ERT* (Cre+) and 4 *$S1P_1^{flox/flox}$* mice that do not carry the *Cre* recombinase (Cre-), respectively).

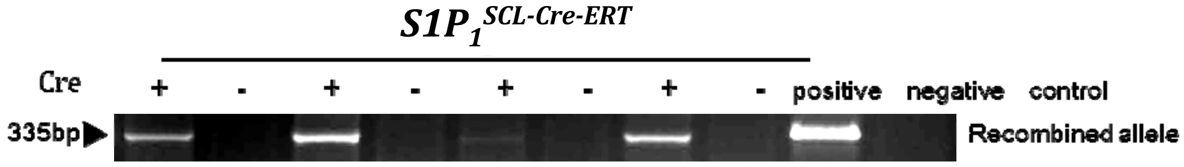

Supplementary Table1: Cell surface expression of PSGL-1 on neutrophils of *$S1P_3^{-/-}$* and *C57Bl6* mice. Data are presented as mean±s.e.m.

Mouse strain	Mice	PSGL-1 positive neutrophils (%)	mean fluorecsence intensity neutrophils PSGL-1 +
C57Bl6	*3*	**96.77 ± 0.28**	**63.67 ± 3.52**
$S1P_3^{-/-}$	*3*	**98.43 ± 0.55**	**71.37 ± 5.66**

Supplementary References

1. Murakami, A.; Takasugi, H.; Ohnuma, S.; Koide, Y.; Sakurai, A.; Takeda, S.; Hasegawa, T.; Sasamori, J.; Konno, T.; Hayashi, K.; Watanabe, Y.; Mori, K.; Sato, Y.; Takahashi, A.; Mochizuki, N.; Takakura, N. *Mol Pharmacol* **2010**, *77*, 704-13

2. Bradley, P. A.; Dack, K. N.; Johnson, P. S.; Skerratt, S. E.; Pfizer Inc., US 20070105909 A1
